# Practical Measurement of Changes in Leg Length Discrepancy After a Myofascial Release on the Thoracolumbar Fascia in Patients With Acute Low Back Pain: A Pilot Study

**DOI:** 10.7759/cureus.29084

**Published:** 2022-09-12

**Authors:** Andreas Brandl, Christoph Egner, Robert Schleip

**Affiliations:** 1 Department of Health and Social Science, DIPLOMA Hochschule, Bad Sooden-Allendorf, DEU; 2 Conservative and Rehabilitative Orthopedics, Department of Sport and Health Sciences, Technical University of Munich, Munich, DEU

**Keywords:** low back pain, osteopathy, manual therapy, leg length discrepancy, myofascial release, thoracolumbar fascia

## Abstract

Background

Recent work has examined an association between leg length discrepancy (LLD) and low back pain (LBP). Myofascial release (MFR) techniques are thought to be frequently applied in the treatment of chronic and acute LBP. The purpose of this study was to evaluate a practical measure of LLD and the feasibility of an MFR technique in a randomised controlled trial (RCT).

Methods

In 12 subjects (seven women and five men) with acute LBP and LLD greater than 3 mm, an MFR technique was performed on the thoracolumbar fascia. At the baseline, after the intervention, and at follow-up, LLD was measured using a cross-line laser and finger-to-floor distance, and the pain was measured with a visual analogue scale (VAS). Patients completed a questionnaire after follow-up to assess the acceptability of the study procedure. The therapist evaluated the methods in terms of their feasibility.

Results

LLD measurement and MFR treatment required little time and few resources. Participants agreed to the study procedure with moderate to high acceptance. The LLD decreased by 5 mm after treatment and by 4 mm at follow-up. The VAS showed a reduction in pain of 17.50 mm at follow-up but not immediately after treatment.

Conclusion

The measurement of LLD is applicable in daily osteopathic practice, but it cannot be assumed to be a valid method for an RCT. Validated methods such as video raster stereography are, therefore, recommended. Comprehensive RCTs to study the effects of MFR intervention on leg length are feasible.

## Introduction

Back pain is a significant health problem in developed countries and is one of the most common reasons for consultations in primary care [1]. The prevalence of non-specific low back pain (LBP) has been reported to be between 30% and 70% in the age group between 18 and 74 years [2]. The remission rate for acute low back pain (aLBP) is believed to be 90% within six weeks, while only 2-7% of patients become chronic [2]. However, these data were collected from studies that defined the duration of pain as the time between the doctor’s visit and the patient’s return to work, rather than studying the actual course of pain [3]. In a meta-analysis of 11 studies (3118 patients), Itz et al. [4] found that the rate of spontaneous recovery of aLBP patients in the first three months was only 33%, while 65% still suffered from LBP one year after the onset of pain. Pengel et al. [5] reported recurrence with renewed incapacity to work in 33% of aLBP cases.

Rannisto et al. [6] found significant correlations between functional leg length discrepancies (LLD) and aLBP in workers at a slaughterhouse. Jeevannavar et al. [7] studied the prevalence of LLD in 245 individuals with non-specific LBP and found that 17% had different leg lengths. Therefore, they recommended that the measurement of LLD should be included in the standard routine examination of LBP patients.

The pelvis is a central part of the kinematic chain between the lower extremities and the spine. An important link in this chain is the sacroiliac joint (SIJ) and its mobility. In a study of six human subjects, Vleeming et al. [8] found motion amplitudes of up to 4.54° in the SIJ. They hypothesised that the abdominal muscles, the abdominal transverse muscle, the internal oblique muscle, and the external oblique muscle exert an anteromedially directed force vector on the anterior superior iliac spine (ASIS). This means that both ASIS regions are pulled towards each other, which increases the pressure on the articular surfaces of the SIJs. However, this biomechanical model only works if a posterior force prevents the two iliac bones from sliding apart in the posterior superior iliac spine (PSIS) region [9]. In this regard, Willard et al. [9] attribute an important role to the thoracolumbar fascia (TLF), along with the multifidus muscle and sacral ligaments. The TLF increases in cross-section, especially in the region caudal to L5 and the os sacrum, and unites with fibres of the sacroiliac ligaments. Other important structures in this power-lock system are the muscles associated with the sacrotuberous ligament, such as the gluteus maximus muscle, in some individuals, the biceps femoris muscle, and the posterior sacroiliac ligaments, which are thought to play the largest role in SIJ stability, themselves [10].

Myofascial release (MFR) techniques are frequently used in manual medicine to restore the optimal length of myofascial tissue structures, improving their function and reducing their pain [11]. Some studies have investigated the efficacy in this regard with conflicting results [12-15]. Ajimsha et al. [11], in a systematic review examining 19 randomised controlled trials (RCTs) of MFR, noted that research on the efficacy of MFR is still in its infancy. To the authors’ knowledge, no other work has examined the relationship between an MFR at the TLF and LLD. This is the first study that has been conducted to evaluate such a relationship.

It is important that an MFR intervention is shown to be feasible and accepted by subjects before full-scale RCTs are conducted. Therefore, the primary objectives of this study were to investigate this and the accuracy and feasibility of LLD measurement, particularly in light of the controversies associated with manual palpation-based measurements [16,17]. The secondary objectives were to collect measurement time point (MT) data and to estimate the treatment effect to calculate sample sizes for subsequent RCTs.

This article was previously posted to the SportRxiv preprint server (https://doi.org/10.31236/osf.io/wvkgr) on May 07, 2021.

## Materials and methods

​Study design overview

A pilot study was conducted with an open pre-post-test single-group design. Measurements were taken before and after the intervention plus one day later, following the SPIRIT (Standard Protocol Items: Recommendations for Interventional Trials) guidelines [18]. The study protocol was registered with the German Registry for Clinical Trials (DRKS00025200) on 26 April 2021. The study was reviewed and approved by the Ethics Committee of the Osteopathic Research Institute in Hamburg, Germany (approval number: 018-05), was conducted in accordance with the Declaration of Helsinki [19], and informed consent was obtained from all participants.

​Setting and participants

The study was conducted in an osteopathic practice in southern Germany. Subjects between 18 and 50 years of age with acute or subacute LBP who achieved a minimum score of 20 on the Oswestry Disability Questionnaire (German version) (ODQ-D) [20] and with pain duration of less than 13 weeks [21] were recruited using a direct approach, a notice board, and distribution of information material in the practice or to acquaintances.

The inclusion criteria were as follows: acute or subacute lumbar back pain, as defined by the European guidelines for the management of aLBP [21]; minimum score of 20 on the ODQ-D; less than 13 weeks pain duration; female or male subjects aged 18 to 50 years; and prone position for 15 minutes must be pain-free for the subjects.

The exclusion criteria were as follows: generally valid contraindications to physiotherapeutic and manual treatments of the lumbar spine and pelvis (i.e. fractures, tumours, infections, and severe cardiovascular and metabolic diseases); pregnancy; rheumatic diseases; scoliotic changes of the lumbar spine; consumption of medications that affect blood coagulation; consumption of muscle relaxants; expression of skin changes (e.g. neurodermatitis, psoriasis, urticaria, and decubitus ulcers); and surgery or scars in the lumbar region between Th12 and S1.

​Intervention

The subjects completed the ODQ-D and were then screened for eligibility by the principal investigator (AB), who then performed the baseline measurements. The subjects received the MFR treatment from a therapist with more than 10 years of professional experience in manual therapy. This was followed by measurements after the intervention and a follow-up one day later.

**Figure 1:**
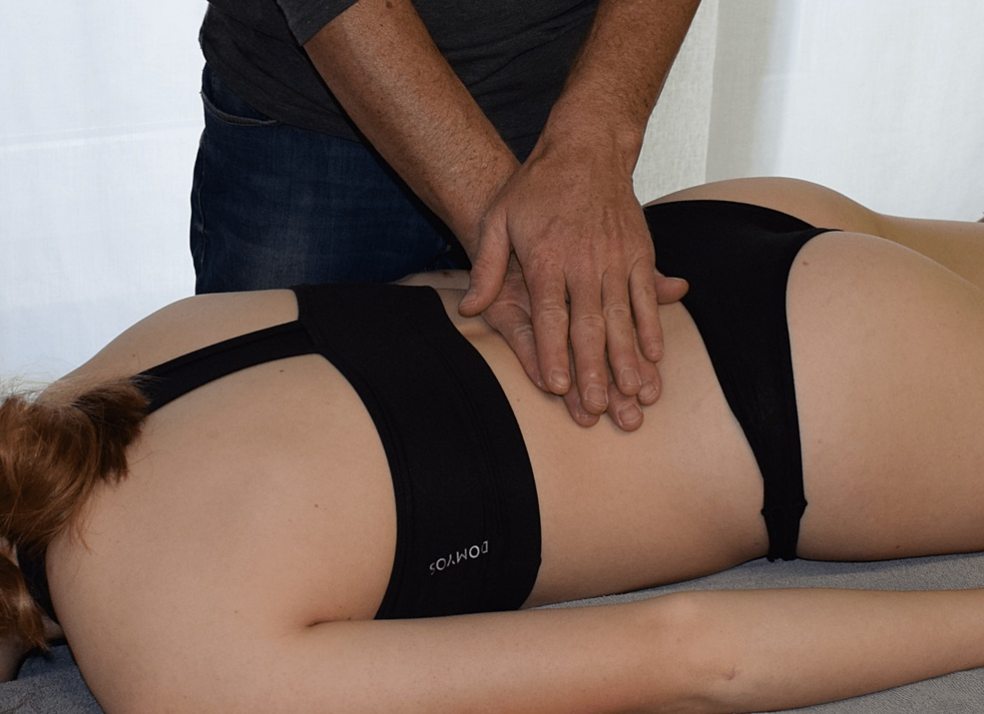
Myofascial release treatment on the thoracolumbar fascia.

Participants received an intervention as described by Chila and O'Connell [22]. The subject is in the prone position with the arms on the sides of the body and the legs parallel to each other. The head is in a neutral position and the face is in a recess in the head section of the therapy table. The patient is sufficiently undressed to access the TLF between Th12 and S1. The therapist stands contralateral to the side to be treated at the level of the subject’s iliac crest (Figure 1). The therapist’s cranial hand, which serves as the palpating hand to evaluate tissue quality, is positioned dorsally immediately adjacent to the lumbar spine, extensively touching the TLF at the level of L1 to L4. The caudal hand duplicates the palpating hand and initiates a direct stretch of the fascia laterally to palpable tissue resistance. The therapist follows the viscoelastic creep effect of the myofascial tissue to initiate further stretching of the TLF [9]. The traction torque applied to the tissue is moderate, ranging from 25 to 35 Nm and acting tangentially laterally to create shear stress between the TLF and the epimysial fascial layer of the erector spinae muscle below the abdominal muscles. The standard force applied during MFR treatment by an experienced therapist was previously measured with a phantom pad placed over a highly sensitive force plate. In addition, he received further training. This was considered sufficient if the blinded therapist was able to perform 30 stroke applications on the force plate, all of which were within this force range and had no outliers. This training was repeated once per day during data collection. The duration of the entire technique is 60 to 90 seconds [22]. However, the crucial factor for the effect is not the amount of time over which the technique is practised, but the occurrence of the MFR effect. Ajimsha et al. [11] define the MFR effect as the restoration of the optimal length of myofascial tissue structures, their functional improvement, and the reduction of pain in them, which can be perceived by the therapist as tissue release.

​Primary outcome measurements

**Figure 2:**
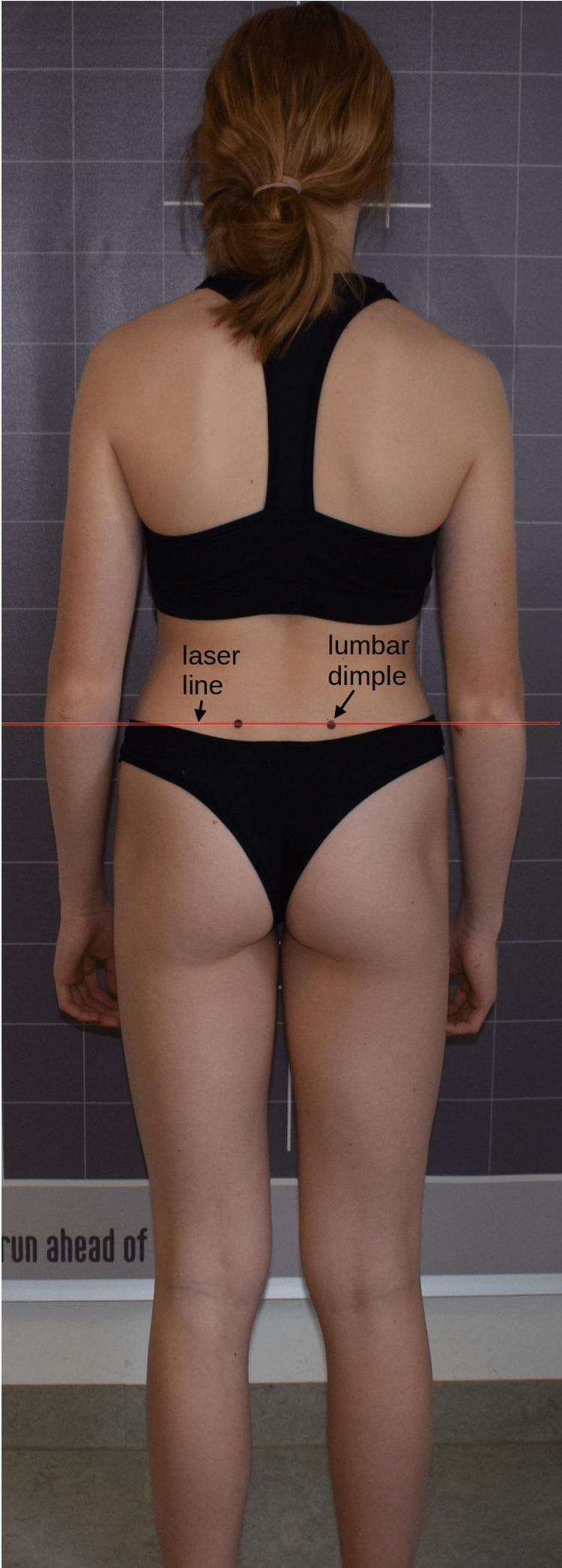
Cross-line laser measurement.

LLD was measured with a cross-line laser (Bosch GLL 2-10 Professional, Robert Bosch Power Tools GmbH, Leinfelden-Echterdingen, Germany). Subjects’ long hair was tied with a hair tie or similar if necessary. The participants then stood barefoot and unclothed except for their underwear with their backs to the measuring device. The lumbar dimples were marked with black dots to determine the height of the presumed PSIS. A horizontal line was then projected through the left dot, parallel to the floor on which the subject's feet were standing (spirit level principle; Figure 2). The LLD is measured as the difference between the laser line and the right PSIS [23]. The measurement accuracy of the laser projection system is 0.5 mm/m. This assessment was used as the primary outcome parameter, based on practicality for the evaluator. The time required for a complete measurement was also recorded.

As another primary outcome parameter, the subjects’ acceptance of the intervention and the measurement procedure was evaluated by a post-treatment survey with a five-point Likert scale.

​Secondary outcome measurements

The LLD was determined using cross-line laser measurement. In addition, the pain was assessed using a visual analogue scale (VAS).

To measure the finger-to-floor distance (FFD), the patients stood with bare feet hip-width apart on a platform to which a millimetre measure was attached [24]. The patients were asked to place their right palm on the back of their left hand and to bend their hips as far as possible with their fingers, arms, and knees extended [25]. The examiner then read the maximum flexion value achieved on the scale. A positive value marked a final position above the stance plane, with a negative value indicating a final position below the stance plane. Ekedahl et al. [24] considered 45 mm to be the minimally detectable change (MDC).

​Statistical analysis

Participant acceptability was assessed using the median, 25% quartile (Q_1_), and 75% quartile (Q_3_). For the secondary outcome parameters, the standard deviation (SD), standard error of the mean (SEM), mean, minimum (min) and maximum (max) values, median, Q_1_, and Q_3_ were obtained for each MT. Missing data from the follow-up measurement (missing completely at random or missing at random) were excluded pairwise. The sample size for future RCTs with two groups and three MTs was calculated with an α-error of 0.05 and a 1-β error of 0.95. The effect size was estimated using the mean differences between the baseline measurements and follow-ups from this pilot study and from a previous paper by Barnes et al. [26]. The effects from an RCT conducted by Wong et al. [15] were also accounted for by the f value from an analysis of variance [27].

LibreOffice Calc version 6.4.7.2 (Mozilla Public License v2.0, The Document Foundation, Berlin, Germany) was used for descriptive statistics. Inferential statistics were carried out with the software R, version 3.4.1 (R Foundation for Statistical Computing, Vienna, Austria). Statistical power was calculated with G*Power (© 1992-2014, Franz Faul, University of Kiel).

## Results

The anthropometric data and baseline characteristics are shown in Table 1.

**Table 1:** Baseline characteristics. SD: standard deviation; n: number; BMI: body mass index; LLD: leg length discrepancy; ODQ-D: Oswestry Disability Questionnaire (German version); VAS: visual analogue scale.

Variable	Mean ± SD	Median (Q_1_ – Q_3_)	Min – Max	n
Age (years)	40.6 ± 10.8	43.4 (41.2 – 47.1)	18.3 – 50.1	12
Gender (m/f)	5/7			12
Height (m)	1.74 ± 0.1	1.73 (1.68 – 1.82)	1.60 – 1.90	12
Weight (kg)	77.2 ± 19.9	73.6 (61.9 – 90.5)	47.0 – 119.0	12
BMI (kg/m^2^)	25.3 ± 5.4	23.6 (22.3 – 27.8)	17.9 – 35.9	12
LLD (mm)	6.1 ± 1.2	6.00 (5.0 – 7.0)	4 – 8	12
VAS (0-100)	28.5 ± 22.7	21.5 (14.5 – 47.8)	0.0 – 75.0	12
ODQ-D (0-100)	25.2 ± 6.1	23.0 (20.0 – 28.5)	20.0 – 36.0	12

​Primary objectives

**Figure 3:**
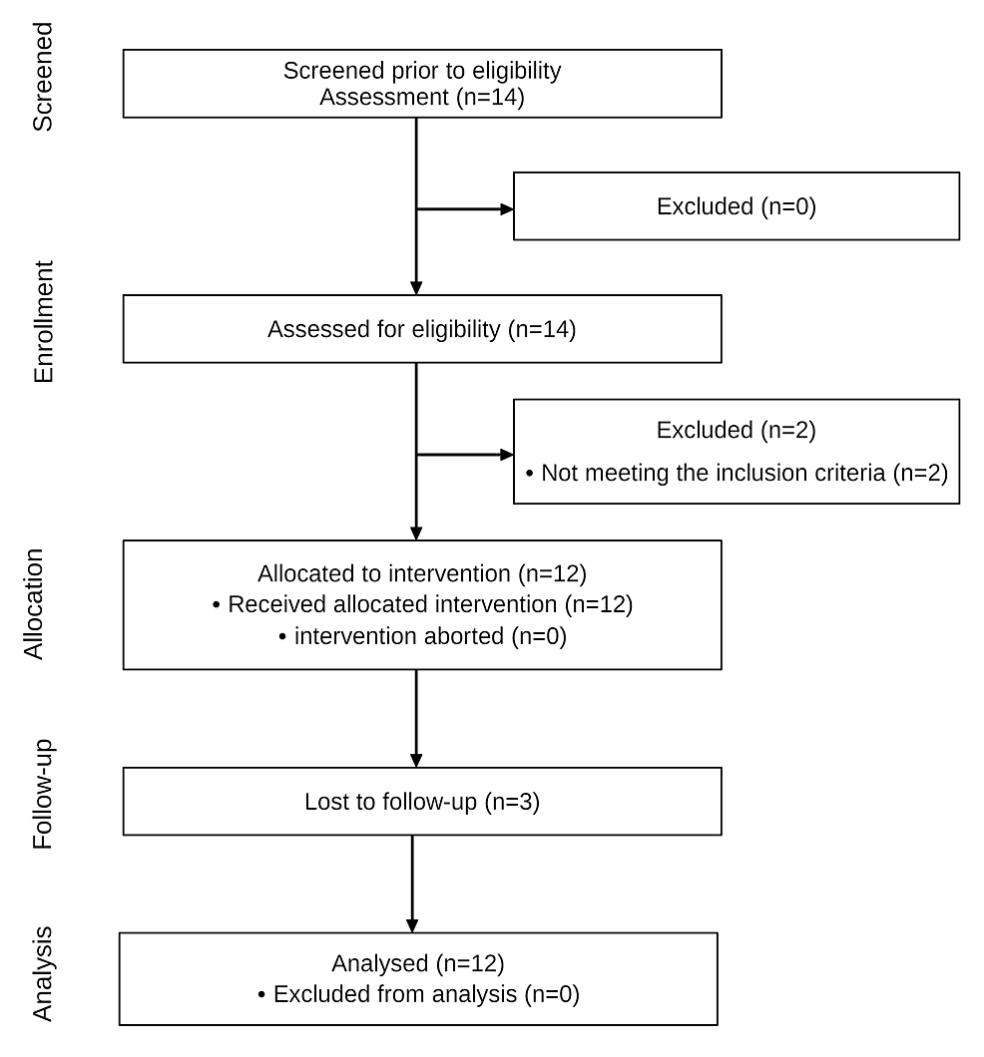
Flow diagram of the study.

Of the 14 subjects screened, 12 met the eligibility criteria and received the intervention (Figure 3); 14% did not meet the eligibility criteria. Three participants dropped out of the study during follow-up. The principal investigator described the time and effort required to perform the cross-line laser measurement as “minimal”.

The results of the follow-up survey of the patients’ acceptance on the five-point Likert scale are shown in Table 2. The participants rated the individual measurements and the manageability of the two examination appointments.

**Table 2:** Follow-up survey of participant acceptance. ODQ-D: Oswestry Disability Questionnaire (German version); VAS: visual analogue scale; FFD: finger-to-floor distance; MFR: myofascial release.

	Median	Q_1_	Q_3_	n
					1. ODQ-D	3	2	4	9
2. VAS	3	2	4	9
3. FFD	5	5	5	9
4. MFR	5	5	5	9
5. Baseline t_1_	5	5	5	9
6. Follow-up t_2_	3	3	4	9

​Secondary objectives

**Figure 4:**
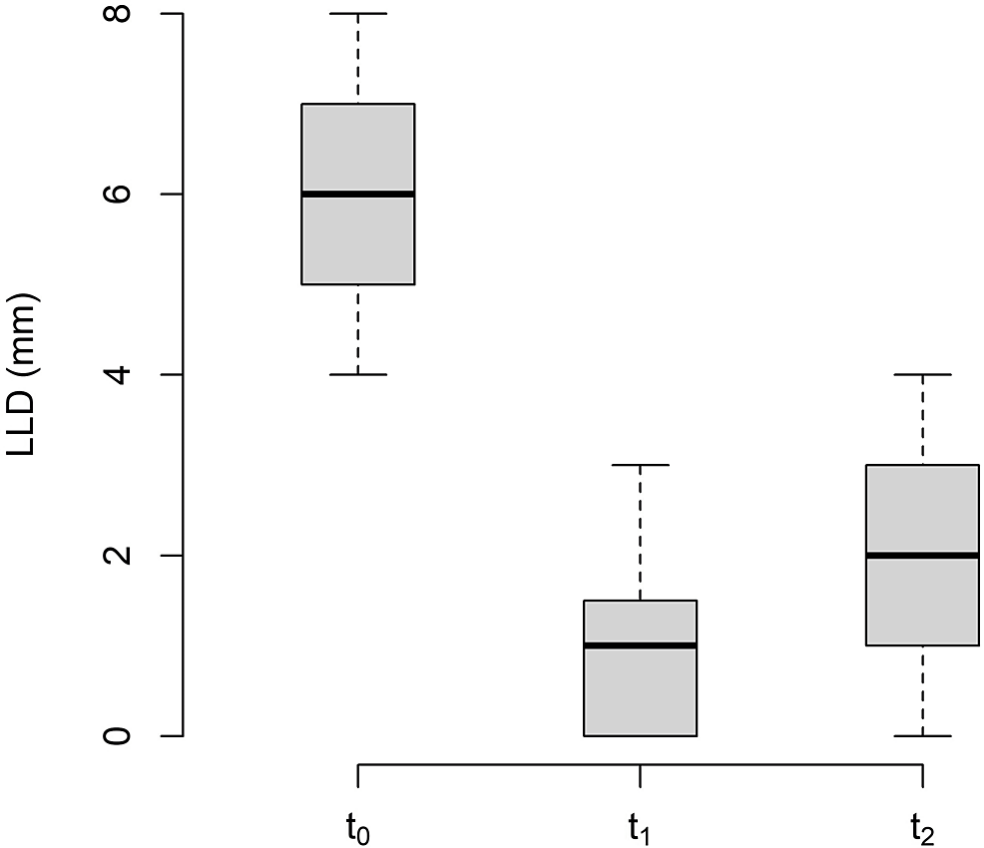
Changes in leg length discrepancy. LLD: leg length discrepancy; t_0_: baseline measurement; t_1_: post-treatment measurement; t_2_: follow-up.

The changes in LLD between the baseline (t_0_), post-treatment (t_1_), and follow-up (t_2_) measurements are shown in Table 3 and Figure 4.

**Table 3:** Descriptive statistics of leg length discrepancy. Values in mm. MT: measurement time point; SD: standard deviation; SEM: standard error of the mean; Q_1_: 25% quartile; Q_3_: 75% quartile; Min: minimum; Max: maximum.

MT	Mean	SD	SEM	Median	Q_1_	Q_3_	Min	Max	n
t_0_	6.08	1.24	0.36	6.00	5.00	7.00	4	8	12
t_1_	0.92	1.00	0.29	1.00	0.00	1.25	0	3	12
t_2_	2.00	1.50	0.92	2.00	1.00	3.00	0	4	9

**Figure 5:**
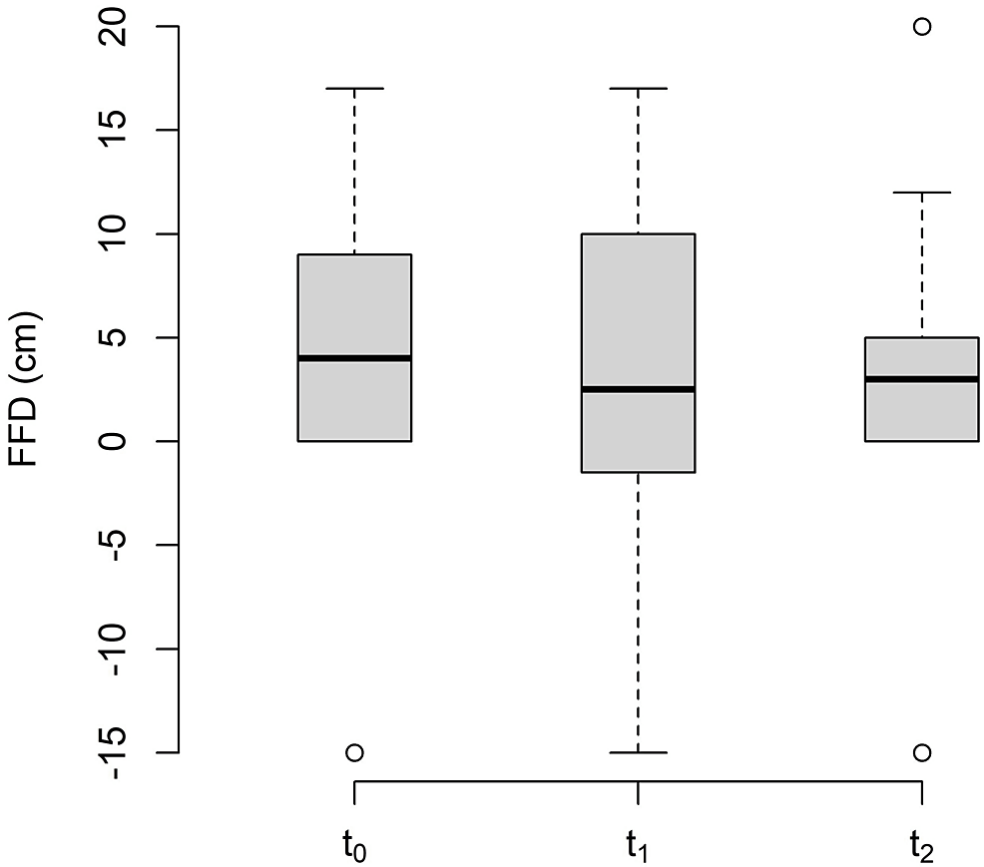
Changes in the finger-to-floor distance. FFD: finger-to-floor distance; t_0_: baseline measurement; t_1_: post-treatment measurement; t_2_: follow-up.

The changes in FFD between the baseline (t_0_), post-treatment (t_1_), and follow-up (t_2_) are shown in Table 4 and Figure 5.

**Table 4:** Descriptive statistics of finger-to-floor-distance. Values in mm. MT: measurement time point; SD: standard deviation; SEM: standard error of the mean; Q_1_: 25% quartile; Q_3_: 75% quartile; Min: minimum; Max: maximum.

MT	Mean	SD	SEM	Median	Q_1_	Q_3_	Min	Max	n
t_0_	4.00	8.06	2.33	4.00	0.00	7.50	-15	17	12
t_1_	2.83	9.28	2.68	2.50	-3.50	9.00	-15	17	12
t_2_	2.11	9.32	2.83	3.00	0.00	5.00	-15	20	9

**Figure 6:**
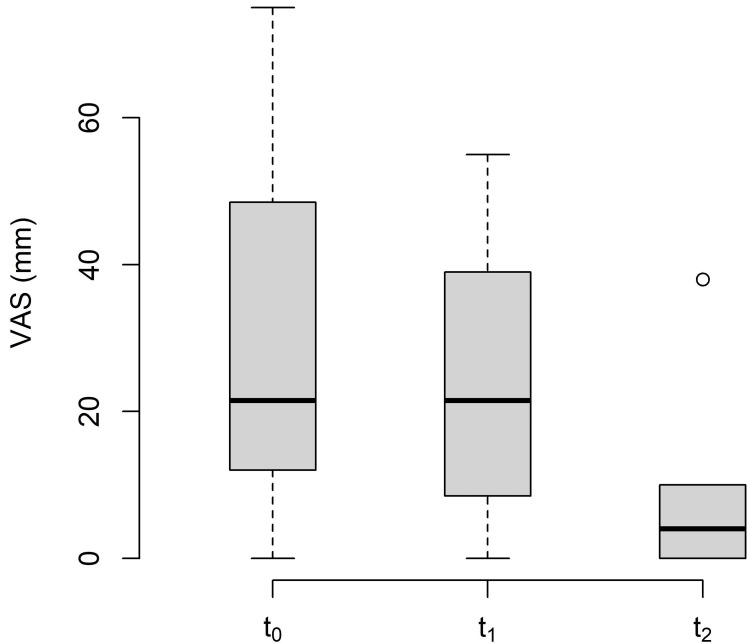
Changes in the visual analogue scale. VAS: visual analogue scale; t_0_: baseline measurement; t_1_: post-treatment measurement; t_2_: follow-up.

The changes in VAS between the baseline (t_0_), post-treatment (t_1_), and follow-up (t_2_) are shown in Table 5 and Figure 6.

**Table 5:** Descriptive statistics of visual analogue scale. Values in mm. MT: measurement time point; SD: standard deviation; SEM: standard error of the mean; Q_1_: 25% quartile; Q_3_: 75% quartile; Min: minimum; Max: maximum.

MT	Mean	SD	SEM	Median	Q_1_	Q_3_	Min	Max	n
t_0_	28.50	22.6	6.55	21.50	14.50	47.75	0	75	12
t_1_	24.25	18.3	5.30	21.50	10.25	36.00	0	55	12
t_2_	7.78	12.0	3.48	4.00	0.00	10.00	0	38	9

The LLD effect size between the baseline measurement and follow-up was f = 0.75. The effect size of the study by Barnes et al. [26] was f = 0.90 and that of the RCT by Wong et al. [15] was f = 0.57. Based on the considerable uncertainty regarding the assumed intervention effects of MFR, we followed the recommendations of Ajimsha and Shenoy [12] and assumed a medium effect size (f = 0.25). Power analysis yielded a total sample size of 44, corresponding to a power of 95% and a significance level of α = 0.05. For future comprehensive RCTs, it is recommended to allow for a 10% loss to follow-up. A sample size of 50 participants (25 in each group) would support this.

## Discussion

​Primary objectives

The results of this study show that RCTs with an MFR treatment group are feasible, but some modifications are needed. The cross-line laser method of measuring LLD achieved a value greater than 5 mm at the post-intervention MT in seven of 12 cases and at follow-up in only two of nine cases. Despite the large difference in mean and the low SD, it cannot be considered a valid method for use under the conditions of an RCT. The reliability is too dependent on the individual experience of the examiner palpating the PSIS for cross-line laser detection. Cooperstein and Hickey [16] found in a systematic review that none of the included studies achieved “moderate” intra- or inter-examiner reliability (κ < 40). However, reliability increases sharply with investigator experience. Lavazza et al. [17] found that only 50% of osteopathic students were able to correctly palpate the PSIS. In contrast, professionals with more than four years of practical experience achieved a detection rate of 84%. Thus, the method can be recommended for daily assessment in an osteopathic or manual therapy practice for therapists with expertise. In this case, it is an uncomplicated, easy-to-use tool for monitoring treatment effects.

The volunteers gave an agreement score of only 3 out of 5 points for the VAS and ODQ-D in the follow-up survey. It is known that many patients fear that grading pain on a scale does not do justice to their individual complex situations [28]. This may influence the acceptance of pain measurement methods. At follow-up, three subjects dropped out. The manageability of a second measurement session one day after the first was rated only 3 out of 5. This needs to be addressed in future RCTs. The LLD, MFR, and manageability of the first appointment were scored as a maximum of five points. It can therefore be assumed that the design and procedure of the pilot study, with the exception of the aforementioned items, are feasible and practicable for future RCTs.

​Secondary objectives

Interpretation of changes in the LLD after treatment is difficult because of the limitations mentioned above. However, seven participants exceeded the MDC immediately after the intervention. Therefore, the mechanisms that might cause this should be discussed. When a unilaterally stiffened TLF is released, the iliac bones can rotate towards symmetry relative to the sacral bone. The MFR investigated in this pilot study could cause an effect as described by Chen et al. [13] for these structures. The tissue stiffness of the middle layer of the TLF, with its connections to the transverse abdominal muscle, could also decrease significantly as a result, initiating an anterior sliding of the pelvic myofascial corset system towards the umbilicus. This would move the ilia towards each other with an anteromedial force vector and allow diarthrotic joint behaviour of the SIJ [8]. The ilium rotates anteriorly and the LLD decreases compared to the baseline measurement. An alternative explanation could be altered neuromuscular behaviour of the erector spinae muscles, causing functional scoliosis and LLD [29]. Proprioception may be affected by adhesions between soft tissue layers, especially the muscle spindles, which could be unilaterally blocked and alter the behaviour of the erector spinae muscles [29]. MFR intervention can influence these mechanisms such that the soft tissue layers regain their ability to glide on each other and unblock the muscle spindles.

The FFD was reduced by 1.17 cm, but this value did not reach the MDC of 4.5 cm [24]. The differences in mean values for each MT were marginal, with a large SD. Ekedahl et al. [24] claimed that the FFD had good validity in patients with aLBP. The authors of the study examined a group of subjects over a period of several months and were able to describe the FFD as a good predictor of a self-documented level of disability. Nevertheless, based on the results of this pilot study, FFD appears to be inappropriate for measuring the short-term effects of MFR on TLF. This is in line with the results of a study by Köck [25], who also investigated the effects of MFR on the FFD and did not find any clinically relevant short-term changes.

The subjects were unable to document a change in subjective pain sensation after MFR using the VAS. Nonetheless, data analysis showed changes both from the baseline measurement to follow-up (-20.72 mm) and between post-intervention measurement and follow-up (-16.47 mm). The MDC was exceeded by six of the nine subjects. These six subjects were all new to the osteopathic practice where the study was conducted and it can be assumed that they received follow-up treatment there. An effect based on the expectation of symptom improvement due to upcoming therapy can therefore not be excluded.

A robust estimate of the intervention effect is needed to calculate the sample size for a comprehensive RCT. To the best of the authors’ knowledge, this was the first study to examine the association between a single MFR treatment on the TLF and associated changes in LLD. Sample size calculations should not be based solely on the effect of a single-arm pilot study with a small sample size. For this reason, previous MFR studies were also included in the estimation of effect size. Unfortunately, limitations (e.g. small sample sizes, incongruent interventions, and methodological limitations) cannot be completely ruled out, so the recommendation of Ajimsha and Shenoy [12] was followed and a medium effect size was assumed. The power analysis then yielded a total sample size of 50, which can be recruited during the daily routine of osteopathic practice.

​Recommendations for future comprehensive RCTs

Cross-line laser measurement with manual palpation of the LLD should be replaced by a validated method, e.g. video raster stereography. Participants’ wishes should be considered when scheduling the follow-up appointment. FFD seems to be inappropriate for measuring the short-term effects of MFR on TLF. The results of the VAS and ODQ-D measurements should be evaluated with caution, taking into account the individual circumstances of the subjects. The sample size for two-arm RCTs should consider only a medium effect and include 25 participants per group.

​Limitations of the study

The potential limitations of this study have been described above. It is important not to interpret the results as evidence of the effectiveness of MFR. There was only one intervention group, which was not blinded. There was no sham control, and the therapist could not be blinded as to which intervention he gave to the subjects. Aside from whether MFR has an effect, it will also be interesting to see how it compares to other techniques and to normal osteopathic or manual therapy. This could be the theme of subsequent RCTs, which may include one or several additional intervention groups.

The limitations should be seen in light of the fact that the primary aim of this pilot study was to prepare and make recommendations for subsequent RCTs.

## Conclusions

The results of this pilot study support the feasibility of subsequent RCTs to investigate the effect of fascia treatment on functional leg length. Cross-line laser measurement with manual palpation does not appear to be sufficiently objectifiable and validatable under the conditions of an RCT to provide usable results regarding functional leg length. Nevertheless, it may be a useful tool for monitoring treatment effects in daily practice. The subjects were positive about the methods in the follow-up survey, with the exception of the pain measurement and follow-up one day after the first appointment.

The FFD measurement should not be included in future short-term intervention studies because it cannot determine the short-term effects of MFR. The VAS score should be considered with caution, taking into account the individual circumstances of the participants and the setting in which the study is conducted. Comprehensive studies would need to include at least 50 volunteers for an intervention and a control group. The results of this feasibility study suggest that comprehensive RCTs with a sufficient number of participants for statistical significance are promising and feasible if a validated measurement method (e.g. video raster stereography) is used in the context of manual therapy practice.

## References

[1] Andersson GBJ (1999). Epidemiological features of chronic low-back pain. Lancet.

[2] Lampert T, Prütz F, Seeling S (2015). Gesundheit in Deutschland. Gesundheitsberichterstattung des Bundes, gemeinsam getragen von RKI und destatis. Robert Koch-Institut.

[3] Bundesärztekammer (BÄK), Kassenärztliche Bundesvereinigung (KBV), Arbeitsgemeinschaft der Wissenschaftlichen Medizinischen Fachgesellschaften (AWMF) (2017). Nationale VersorgungsLeitlinie Nicht-spezifischer Kreuzschmerz - Langfassung, 2. Auflage. Medizinischen Fachgesellschaften.

[4] Itz CJ, Geurts JW, van Kleef M, Nelemans P (2013). Clinical course of non-specific low back pain: a systematic review of prospective cohort studies set in primary care. Eur J Pain.

[5] Pengel LH, Herbert RD, Maher CG, Refshauge KM (2003). Acute low back pain: systematic review of its prognosis. BMJ.

[6] Rannisto S, Okuloff A, Uitti J, Paananen M, Rannisto PH, Malmivaara A, Karppinen J (2015). Leg-length discrepancy is associated with low back pain among those who must stand while working. BMC Musculoskelet Disord.

[7] Jeevannavar JS, Ganesh GA, Jeevannavar SS (2018). Prevalence of leg length discrepancy in persons with non-specific low back pain. Indian J Physiother Occup Ther.

[8] Vleeming A, Buyruk HM, Stoeckart R, Karamursel S, Snijders CJ (1992). An integrated therapy for peripartum pelvic instability: a study of the biomechanical effects of pelvic belts. Am J Obstet Gynecol.

[9] Willard FH, Vleeming A, Schuenke MD, Danneels L, Schleip R (2012). The thoracolumbar fascia: anatomy, function and clinical considerations. J Anat.

[10] Panjabi MM (2006). A hypothesis of chronic back pain: ligament subfailure injuries lead to muscle control dysfunction. Eur Spine J.

[11] Ajimsha MS, Al-Mudahka NR, Al-Madzhar JA (2015). Effectiveness of myofascial release: systematic review of randomized controlled trials. J Bodyw Mov Ther.

[12] Ajimsha MS, Shenoy PD (2019). Improving the quality of myofascial release research - a critical appraisal of systematic reviews. J Bodyw Mov Ther.

[13] Chen YH, Chai HM, Shau YW, Wang CL, Wang SF (2016). Increased sliding of transverse abdominis during contraction after myofascial release in patients with chronic low back pain. Man Ther.

[14] Laimi K, Mäkilä A, Bärlund E (2018). Effectiveness of myofascial release in treatment of chronic musculoskeletal pain: a systematic review. Clin Rehabil.

[15] Wong KK, Chai HM, Chen YJ, Wang CL, Shau YW, Wang SF (2017). Mechanical deformation of posterior thoracolumbar fascia after myofascial release in healthy men: a study of dynamic ultrasound imaging. Musculoskelet Sci Pract.

[16] Cooperstein R, Hickey M (2016). The reliability of palpating the posterior superior iliac spine: a systematic review. J Can Chiropr Assoc.

[17] Lavazza C, Milano V, Abenavoli A, Maggiani A (2018). How type and number of training sessions influence the reliability of palpation. J Bodyw Mov Ther.

[18] Chan AW, Tetzlaff JM, Gøtzsche PC (2013). SPIRIT 2013 explanation and elaboration: guidance for protocols of clinical trials. BMJ.

[19] World Medical Association (2013). World Medical Association Declaration of Helsinki: ethical principles for medical research involving human subjects. JAMA.

[20] Gaul C, Mette E, Schmidt T, Grond S (2009). ODQ - Oswestry Low Back Pain Disability Questionnaire - Deutsche fassung. BioMed Res Int.

[21] van Tulder M, Becker A, Bekkering T (2006). European guidelines for the management of acute nonspecific low back pain in primary care. Eur Spine J.

[22] Chila AG, O’Connell JA (2010). Foundations of Osteopathic Medicine. Foundations of Osteopathic Medicine.

[23] Bruzek R (2006). Messgeräte. Leitfaden Gelenkmessung.

[24] Ekedahl H, Jönsson B, Frobell RB (2012). Fingertip-to-floor test and straight leg raising test: validity, responsiveness, and predictive value in patients with acute/subacute low back pain. Arch Phys Med Rehabil.

[25] Köck J (2015). Änderung der Viskoelastizität im Bereich des lumbalen Weichteilgewebes durch eine osteopathische Technik an der Fascia thoracolumbalis und deren Einfluss auf die Rumpfflexion im Stand. Dresden International University.

[26] Barnes MF (1997). Efficacy study of the effect of a myofascial release treatment technique on obtaining pelvic symmetry. J Bodyw Mov Ther.

[27] Lenhard W, Lenhard A (2017). Computation of effect sizes. Psychometrica.

[28] Karner JJ (2012). Die Abbildung chronischer Schmerzen anhand von validierten Fragebögen. Die Abbildung chronischer Schmerzen anhand von validierten Fragebögen - Eine qualitative Studie bei älteren Patienten mit chronischen Schmerzen der Halswirbelsäule..

[29] Stecco A, Gesi M, Stecco C, Stern R (2013). Fascial components of the myofascial pain syndrome. Curr Pain Headache Rep.

